# Double Antiresonance Fiber Sensor for the Simultaneous Measurement of Curvature and Temperature

**DOI:** 10.3390/s21237778

**Published:** 2021-11-23

**Authors:** Diana Pereira, Jörg Bierlich, Jens Kobelke, Marta S. Ferreira

**Affiliations:** 1 i3N & Department of Physics, University of Aveiro, Campus Universitario de Santiago, 3810-193 Aveiro, Portugal; dsap@ua.pt; 2 Leibniz Institute of Photonic Technology IPHT, Albert-Einstein-Str. 9, 07745 Jena, Germany; joerg.bierlich@leibniz-ipht.de (J.B.); jens.kobelke@leibniz-ipht.de (J.K.)

**Keywords:** antiresonant optical fiber, hollow square core fiber, curvature sensing, temperature sensing

## Abstract

Antiresonant hollow core fibers (ARHCFs) have gained some attention due to their notoriously attractive characteristics on managing optical properties. In this work, an inline optical fiber sensor based on a hollow square core fiber (HSCF) is proposed. The sensor presents double antiresonance (AR), namely an internal AR and an external AR. The sensor was designed in a transmission configuration, where the sensing head was spliced between two single mode fibers (SMFs). A simulation was carried out to predict the behaviors of both resonances, and revealed a good agreement with the experimental observations and the theoretical model. The HSCF sensor presented curvature sensitivities of −0.22 nm/m^−1^ and −0.90 nm/m^−1^, in a curvature range of 0 m^−1^ to 1.87 m^−1^, and temperature sensitivities of 21.7 pm/°C and 16.6 pm/°C, in a temperature range of 50 °C to 500 °C, regarding the external resonance and internal resonance, respectively. The proposed sensor is promising for the implementation of several applications where simultaneous measurement of curvature and temperature are required.

## 1. Introduction

The development of photonic crystal fibers (PCFs) has revolutionized the sensing field. The unique capability that PCFs have on manipulating some of the optical properties, such as birefringence, nonlinearities, and dispersion [1,2,3], have made them an exquisite topic of interest in the scientific community. Among the category of the PCFs, hollow core PCFs (HC-PCFs) can be highlighted, which have attracted great interest due to their aptitude on guiding light with relatively low-loss [2,4]. The recent breakthrough of the antiresonant hollow core fiber (ARHCF), a new category of HC-PCF, allowed not only to overcome several difficulties in the telecommunication area, but also to fully exploit the resonance behavior in new sensing applications, specifically in the optofluidics area [4,5,6] and in the biomedical and biochemical fields [4,7,8]. The ARHCF, whose light guidance relies on the principle of antiresonant reflecting optical waveguide (ARROW) [9,10], has been subject of scrutiny due to the numerous advantages it holds, such as ultralow loss and dispersion [4,11,12,13,14], reduced nonlinearities [13,15], ultrashort pulse delivery [13,16,17,18], and a broad bandwidth [11,12,19]. With the perspective of a potential sensing implementation, the ARHCF has been used in the detection of chemical substances [20,21,22], and in the measurement of physical and mechanical properties [23,24,25,26,27,28].

Curvature and temperature sensors find themselves crucial in many current applications, specifically in fields of mechanical and civil engineering [29,30], structural health monitoring [29,31], astrophysics [31], industry [32], and medicine [33,34]. With the prospect of a wide practicability, several temperature and curvature sensors based on ARHCF have been designed and further developed in the past years. For instance, Liu et al. [35] proposed a temperature ARHCF sensor based on a HCF spliced between two single mode fibers (SMF), in a transmission configuration, with a sensitivity of 33.4 pm/°C. Additionally, a curvature sensor has been reported by Herrera-Piad et al. [36] using a capillary hollow core fiber assembled between two SMFs. The sensing structure exhibited a sensitivity of 1.6 dB/m^−1^ within a curvature range up to 2.14 m^−1^. Moreover, a hybrid sensor for the simultaneous measurement of temperature and curvature has been proposed by Cheng et al. [37], with the ARHCF being spliced between two SMFs. The acquired sensitivities for the temperature and curvature were 25.76 pm/°C and −4.28 dB/m^−1^, respectively. Further sensors have already been developed to measure these parameters [38,39].

In this work, the use of a newly designed ARHCF is demonstrated to simultaneously measure curvature and temperature. The fiber used in this study is hollow square core fiber (HSCF), due to the particular square shape of the core, and incorporates two resonance mechanisms, namely, an internal resonance (IR) and external resonance (ER). The sensing structure was attained by resorting to a simple configuration SMF-HSCF-SMF. The purpose of the work relies on using both resonance mechanisms, intrinsically inherent to the same sensing head, to monitor the two physical parameters, a feature that, to the best of our knowledge, has not been reported yet. 

## 2. Fiber Geometry

**Figure 1:**
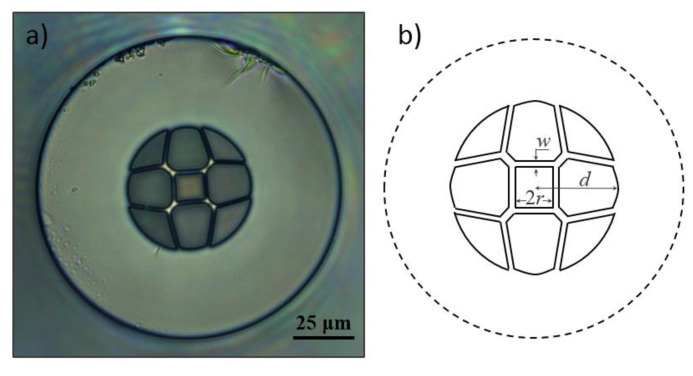
(**a**) Microscopic (400×) picture of the HSCF cross-section. (**b**) Geometrical scheme of the HSCF.

Figure 1a presents a microscopic picture of the HSCF implemented in this work, and Figure 1b shows a scheme of the cross-section model of the fiber structure. The fiber was developed at the Leibniz Institute of Photonic Technology, in Germany. It is characterized by having a hollow core of ~11 μm size (2*r*) in a squared shape. Surrounding the core, there are silica strands of ~1.7 μm of thickness (*w*) conjugated with four capillaries diametrically opposed. Furthermore, the HSCF presents four identical air petal shape structures, intercalated with four interstices. The internal radius of the fiber, where all the air structures are located, is of ~26 μm (*d*), and the external radius is ~62.5 μm. All components of the HSCF were fabricated from synthetic high-purity silica tubes (F300, Heraeus Quarzglas GmbH & Co. KG, Hanau, Germany). The fiber was coated with a single UV acrylate layer during the fabrication. The manufacturing process as well as the formation of the specific fiber microstructure are described elsewhere [40].

## 3. Principle of Operation

**Figure 2:**
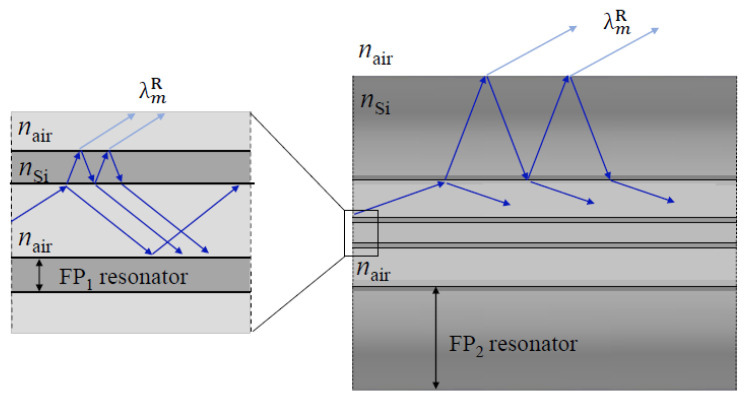
Scheme of the optical paths of light in the internal AR guiding process, which occurs in the core area, and the external AR, that occurs in the outer cladding section.

The light guidance in the HSCF relies on the antiresonance reflection (AR), making this fiber inherent to the ARHCF class. The AR principle can be described by the ARROW model, which expresses the optical fiber as an array of high and low refractive index layers, and where the former act as Fabry-Perot resonators [41,42]. The wavelengths that do not obey the resonance condition are reflected within the resonator, and thus, they propagate along the fiber core. As for the wavelengths that obey the resonance condition, the high index layers become translucent to light, allowing it to escape from the FP resonator, being posteriorly lost. The resonance wavelengths can be attained by the following expression [43]:

(1) $$\lambda_{m}^{R}=\frac{2w\sqrt{n_{\mathrm{Si}}^{2}-n_{\mathrm{air}}^{2}\sin^{2}\alpha }}{m},$$

where $n_{\mathrm{Si}\;}$ and $n_{\mathrm{air}\;}$are the refractive indices of the waveguide material (silica) and air, respectively, *m* is the resonance order, and α is the incident angle. For the AR guidance, the reflections present glancing angles; therefore, sinα can be approximated to the unit, leading to a simplification in the expression. In the HSCF, there are two distinctive AR guidance mechanisms, namely an internal AR, where light is trapped in the core and surroundings of it, and the external AR, wherein light remains confined in the outer cladding region. Figure 2 illustrates the light propagation in the fiber that originates these two ARs. The light that propagates in the air core, when encountering the interface between the core and the silica strands, will be both reflected, thus remaining in the air core, and refracted to the silica strands. The silica strands will be acting as an FP resonator, which means that for the AR wavelengths, light will be reflected within the resonator and refracted back to the core. As for the resonance wavelengths (internal resonance, IR), light will escape the resonator and will leak to the air structures, being further lost as it propagates [40]. The second AR guidance occurs when light leaks from the core, and thus, it propagates in the petal and interstitial air structures that surround the core. There, light will also be refracted to the outer silica cladding section, which, in turn, will also act as an FP resonator, meaning that the resonance wavelengths (external resonance, ER) will leak out of the fiber.

To further understand the AR principle, particularly the internal AR, it is necessary to comprehend the propagation of the fundamental mode (HE_11_) as well as its intrinsic properties. Considering the perturbation theory model, encountered in [44], the effective refractive index of a propagating mode is described by the following expansion:

(2) $$n_{\mathrm{eff}}=n_{\mathrm{air}}\left(1-a\sigma^{2}-b\sigma^{3}-c\sigma^{4}+id\sigma^{4}\right),$$

where *σ* is denoted as the perturbation parameter and is inversely proportional to the core wavenumber, $k_{\mathrm{air}}$, and the core radius r. The core wavenumber is related with the vacuum wavenumber, $k_{0}$, through $k_{\mathrm{air}}=k_{0}n_{\mathrm{air}}$. The coefficients a,b, c, and d are real numbers and can be described by the real coefficients of the radial wavenumber, $k_{1},k_{2}$, and the complex coefficient, $k_{3}.$ With this, it is possible to establish the following correlations [44]:

(3) $$a=\frac{k_{1}^{2}}{2}=\frac{j_{m-1,n}^{2}}{2},$$

(4) $$b=k_{1}k_{2}=\frac{j_{m-1,n}^{2}\left(\epsilon +1\right)\cot\left(\phi \right)}{2\sqrt{\epsilon -1}},$$

(5) $$c=\frac{k_{1}^{4}}{8}+\frac{k_{2}^{2}}{2}+k_{1}ℜ\left(k_{3}\right)=\frac{j_{m-1,n}^{4}}{8}c_{0}+\frac{j_{m-1,n}^{2}}{2}c_{1},\;$$

(6) $$d=-k_{1}ℑ\left(k_{3}\right)=\frac{j_{m-1,n}^{3}}{2}\frac{\left(\epsilon^{2}+1\right)}{\epsilon -1}\left(1+\cot{\left(\phi \right)}^{2}\right),\;$$

with:

(7) $$c_{0}=1-\frac{\left(\epsilon -1\right)}{m}\cot^{2}\left(\phi \right),$$

(8) $$c_{1}=\frac{\left(4-2m\right){\left(\epsilon +1\right)}^{2}}{4\left(\epsilon -1\right)}\cot^{2}\left(\phi \right)-m.$$

The variable $j_{m-1,n}$ represents the *n*th root of the Bessel function m−1, for the hybrid mode HE_m,n_, ϕ is the accumulated phase between two consecutive reflections of a light ray in the silica glass structure, and the parameter $\epsilon ={\left(n_{\mathrm{Si}}/n_{\mathrm{air}}\right)}^{2}$. Developing the set of equalities from Equations (2)–(8), one concludes that the effective refractive index can be given by:

(9) $$n_{\mathrm{eff}}=n_{\mathrm{air}}-\frac{j_{m-1,n}^{2}}{2k_{o}^{2}n_{\mathrm{air}}r^{2}}\;\left(1-\frac{\left(\epsilon -1\right)\cot\left(\phi \right)}{\sqrt{\epsilon -1}k_{0}n_{\mathrm{air}}r}\right)+i\left(\cot{\left(\phi \right)}^{2}+1\right)\frac{j_{m-1,n}^{3}(\epsilon^{2}+1)}{2\left(\epsilon -1\right)k_{o}^{4}n_{\mathrm{air}}^{3}r^{4}}.$$

**Figure 3:**
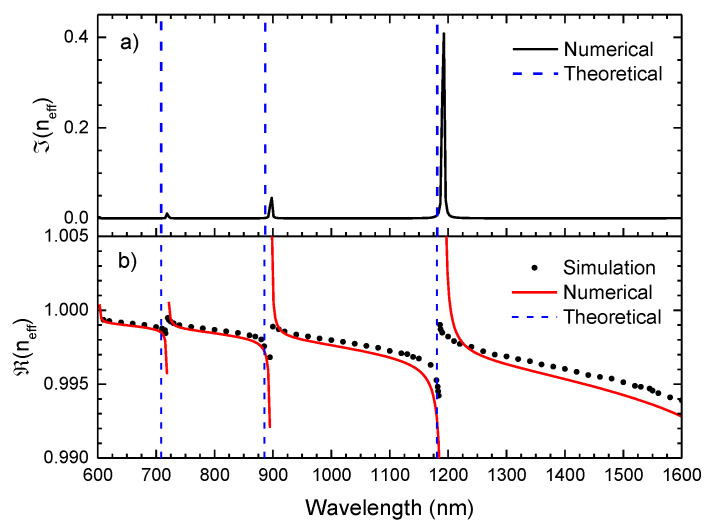
(**a**) Imaginary effective refractive index with wavelength obtained from Equation (9) (Numerical). (**b**) Real component of the effective refractive index with a wavelength attained by the COMSOL Multiphysics (Simulation) and by Equation (9) (Numerical). The expected frequency of the IR is represented by dash lines, according to Equation (1).

Based on the analytical expression in Equation (9), a complex effective refractive index is attained, where the real component describes the spectral tendency of the leaky mode effective index, while the imaginary part is associated with the losses inherent to the mode propagation. Figure 3 exhibits the imaginary and real part of the effective refractive index of the HE_11_ mode, attained by using Equation (9). It is also presented the simulated profile of the real part of the effective refractive index of the HSCF in the spectral range of the visible and infrared, resorting to the COMSOL Multiphysics. The COMSOL Multiphysics simulation (version 5.6) was carried out over a wavelength range between 600 nm and 1600 nm, in steps of 1 nm near the resonance wavelengths and steps of 10 nm in the regions away from these. The Sellmeier equation was used to estimate the refractive index of silica for each wavelength.

Analysing the real part of the refractive index, a good agreement between the numerical curve and the simulated data is attained, validating, therefore, the use of Equation (9) to describe the resonance within the HSCF. The asymptotic tendency of the refractive index real component should also be highlighted. In fact, this behavior is highly characteristic in ARHCFs, where the asymptotic curves tend to a specific wavelength, that is, the resonance wavelength. This fact is also proved by the behavior of the imaginary component of the refractive index, where high intensity peaks are notoriously located in the same frequencies as the asymptotic wavelength. To further corroborate this explanation, the theoretical values of the resonance wavelengths, calculated by Equation (1), were also represented in Figure 3. A good match between the simulated and the theoretical resonance is perceived, although there is a small shift between the theoretical and numerical resonance wavelengths. This can be justified by the simplified geometrical approximation used in the numerical model. However, in an overall perspective, the numerical equation describes this fiber with a relatively good accuracy.

**Figure 4:**
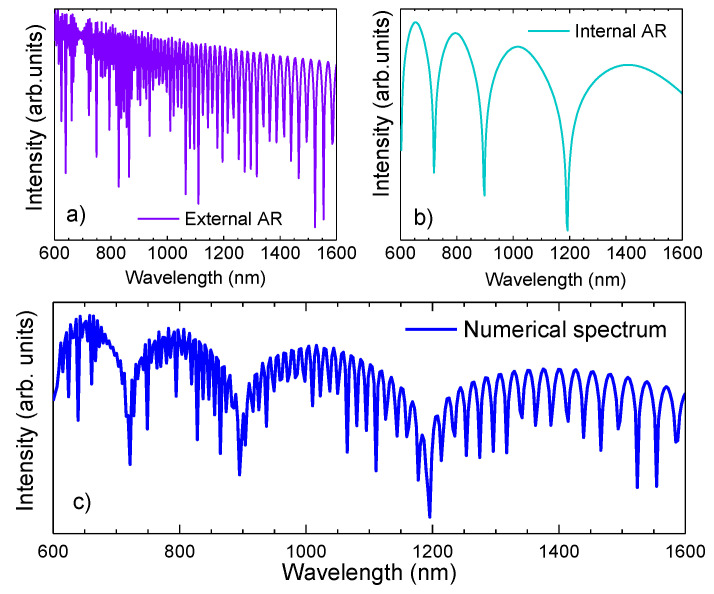
(**a**) Numerical solution of the external AR spectrum. (**b**) Numerical solution of the internal AR spectra. (**c**) Numerical solution of the transmission spectrum of the HSCF.

Moreover, the use of Equation (9) is not limited to the IR, with its usage being capable of also predicting the ER. Notice that the IR is resultant of the core mode leakage, while the ER is induced by the leakage of the cladding mode. Therefore, instead of analyzing the HE_11_ mode, one has to analyze the first cladding mode, that is, the hybrid mode HE_12_. Furthermore, the parameters *r* and *w*, which were considered to be the core radius and the silica strands thickness, will change since in the external resonance the fiber is comparable to a capillary with a core radius of *d* and thickness equal to the difference between the fiber radius and the core radius. With the conjecture of a complex effective refractive index, where the imaginary part is associated with the losses subjected to the mode propagation, it is possible to attain the profile loss associated with the HSCF, by considering that the major factor that induces losses in the HSCF and the significant diminishing of the optical power is the confinement loss (*CL*) of the propagating mode. Since in the HC-PCFs, the light is guided within air, loss factors such as absorption and the Rayleigh scattering are too small [2,45]; therefore, they were disregarded. The confinement loss is as follows [46]:

(10) $$CL=\frac{40\pi ℑ\left(n_{\mathrm{eff}}\right)}{\ln\left(10\right)\lambda },$$

where $ℑ\left(n_{\mathrm{eff}}\right)$ was retrieved from the imaginary component of Equation (9); thus, one can estimate the transmission windows that are formed by both the external and internal AR propagations, and consequently, the expected transmission spectrum of the HSCF, considering the total modal losses inherent to the HSCF length (*l*). Notice that to attain the expected transmission profile, a normalization was carried out to the numerical results to better perform a comparison with the experimental results that will be described further ahead. In Figure 4a,b, the transmission curves attained for each AR mechanism are represented, as well as the combined signal (Figure 4c).

## 4. Results and Discussion

### 4.1. Sensor Design and Experimental Setup 

**Figure 5:**
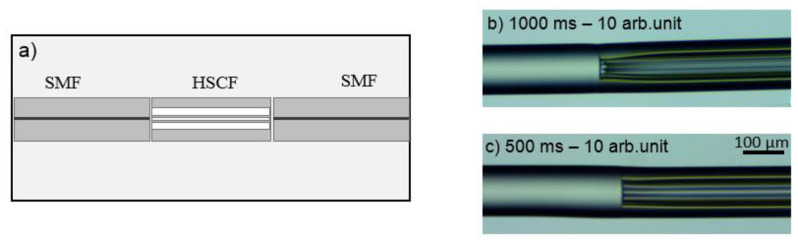
(**a**) Schematic representation of the sensor fabrication, wherein the HSCF is spliced between two SMFs and (**b**,**c**) longitudinal view of the splicing area for different arc discharge times.

The layout of the sensor used in this work was based on a transmission configuration, where the HSCF was spliced between two segments of SMF, as can be seen in Figure 5a. The splicing process was executed using a Fujikura 40S splicer, which was operated in manual mode, that is, the alignment between fibers was personalized by the user. Given the geometry of the HSCF and the fragility of its microstructures, it was necessary to adjust the parameters of the splicing program so that one could attain an equilibrium between the splicing strength and the integrity of the HSCF structure. To keep the cohesion of the microstructures intact, the arc was applied mainly on the SMF. In addition, to avoid compromising the splicing resistance, it was necessary to adjust the arc discharge power and duration. Figure 5b,c show the influence of the splicing parameters in the HSCF structure. Longer arc discharge times give rise to a collapsing of the external cladding as well as a damage in the internal structure. Therefore, values of 10 arbitrary units (arb. units) and 500 ms were used for the arc power and duration, respectively (for comparison purposes, in an automatic splice, these parameters are set to 20 arb. units and 2000 ms). Several sensors were produced, and their lengths were measured using a caliper.

**Figure 6:**
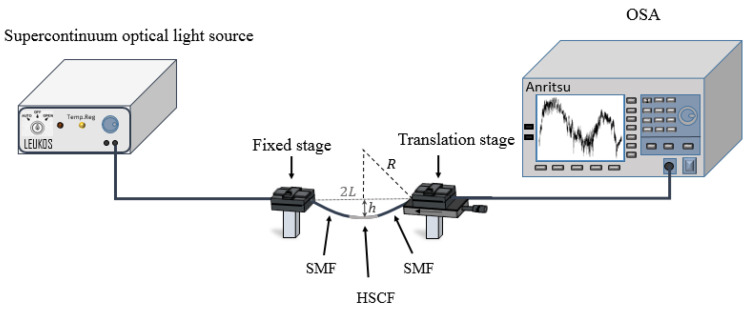
Scheme of the experimental setup used for the curvature measurements.

The sensor was placed into a transmission configuration, where a supercontinuum optical light source (LEUKOS SAMBA 450) and an optical spectrum analyzer (Anritsu MS9740A), with a resolution of 0.2 nm, were used, as depicted in Figure 6. 

For the curvature measurements the sensor was glued in a fixed stage and in a translation stage. The controlled movement of the last towards the direction of the fixed stage, induces a bending on the sensor. The curvature can be determined by the following expression [29]:

(11) $$C=R^{-1}=\frac{2h}{h^{2}+L^{2}}\;,\;$$

where 2*L* is the distance between the points where the sensor is fixed, *h* is the height of the sensor center to the horizontal plane, and *R* is the radius of bending. Additionally, temperature measurements were carried out by resorting to a similar configuration as in Figure 6, although the temperature variations were accomplished by resorting to a custom designed tubular furnace. The furnace temperature was controlled by a thermocouple with a resolution of 1 °C.

### 4.2. Spectral Characteristics 

**Figure 7:**
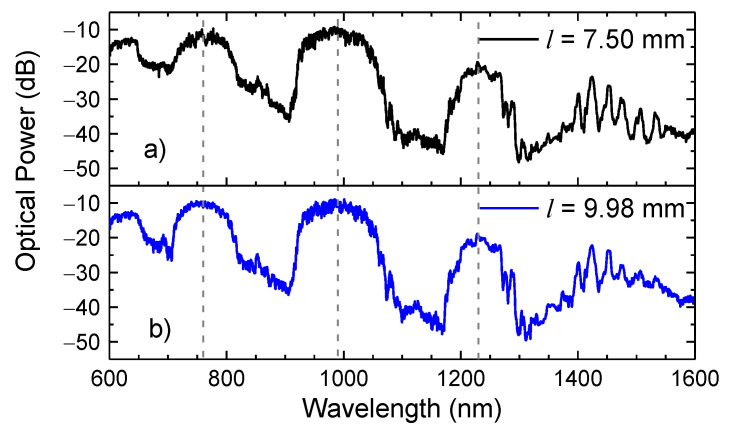
Transmission spectra of a HSCF sensor with lengths of (**a**) 7.50 mm and (**b**) 9.98 mm. The dash lines represent the central wavelength of each transmission band.

To access an overall perspective of the sensor length influence on the measurements, it was necessary to appraise the spectral response of sensors with distinct sizes. Therefore, two sensors with lengths of 7.50 mm and 9.98 mm were monitored in a broadband ranging from the visible to the infrared windows (600 nm–1600 nm). Figure 7 presents the transmission spectra attained. 

From a first analysis, one can see that sensors’ spectra are characterized by large transmission bands centered at 760 nm, 990 nm, and 1230 nm. In between there are large depression bands, which originate from the IR and whose wavelengths satisfy the condition established in Equation (1). Aside from the transmission bands, there is another band, which begins at ~1270 nm, where it is possible to identify several peaks, with a smaller free spectral range, which result from the external AR guidance. The spectra of both 7.50 mm and 9.98 mm sensors are quite identical, presenting the same transmission bands and the intensity peaks in the exact frequencies, meaning that the length of the sensor does not appear to influence on the transmission spectrum nor the resonance mechanisms that are inherent to it. This was already expected by Equation (1). 

**Figure 8:**
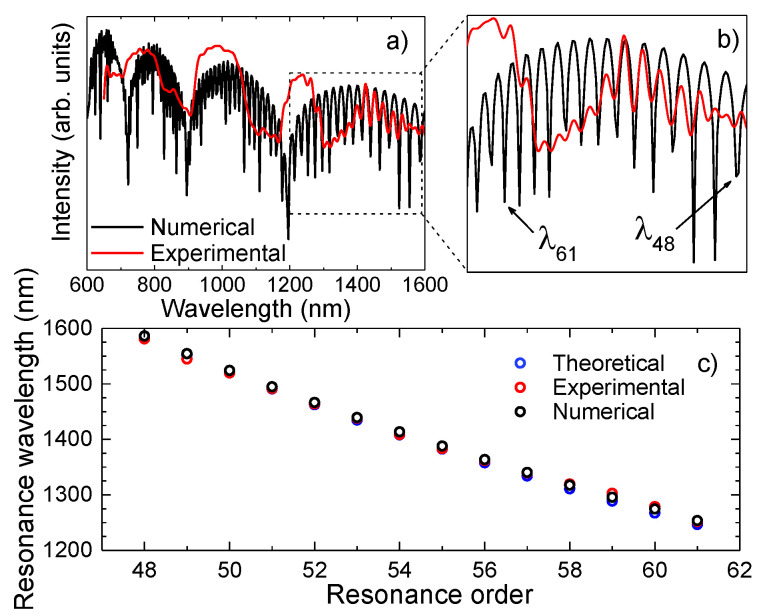
(**a**) Representation of the simulated and experimental transmission spectrum of the 7.50 mm HSCF. (**b**) Amplification of the transmission spectra in the range of 1200 nm−1600 nm, where the external AR modulation is observable. (**c**) Values of the ER dips attained from the numerical and experimental spectrum, and the theoretical values from Equation (1).

The attained experimental and simulated spectra of a 7.5 mm long sensor are represented in Figure 8a. The similarity between the two is quite notorious. In fact, by observing the zoom in of the transmission spectrum (Figure 8b), between 1200 nm and 1600 nm, a good agreement between the ER peaks is perceptible. It was possible to determine the resonances from the 48th order to the 61st order, by using Equation (1). The differences observed between the high frequency component attained experimentally and the one obtained numerically can be due to different effects, such as losses due the splicing procedures, surface imperfections or mode mismatch, which were not considered in the numerical model. Figure 8c presents the resonance wavelengths retrieved from the experimental spectrum, determined by Equation (1), and by the numerical solution. Although the results are similar, for higher resonance orders, there is a slight deviation. This can be partly justified by the impact of the internal AR guiding that attenuates this phenomenon, therefore leading to a higher difficulty in distinguishing the ER. As for the IR, the HSCF spectrum presents resonance bands and not dips, as one would expect. This could be justified by the slight variations on the thickness of each strand that surround the core, combined with possible fluctuations along the HSCF length, causing a significant deviation on the resonance wavelength, and, therefore, the creation of bands [47]. For instance, considering a variation of 0.1 μm in the strands thickness, according to Equation (1), a shift of 70 nm in the transmission spectrum will be originated. Still, one must notice that the expected resonance wavelengths are within the resonance bands. Furthermore, the amplitude decrease of the AR bands is notorious for higher wavelengths. This can be an indication that the core modes are not so well confined within the core region, and the coupling of light to the cladding region will be favored. Thus, the external AR will be further stimulated, leading to a notorious modulation on the transmission spectrum of the phenomenon at higher wavelengths (1200 nm–1600 nm).

**Figure 9:**
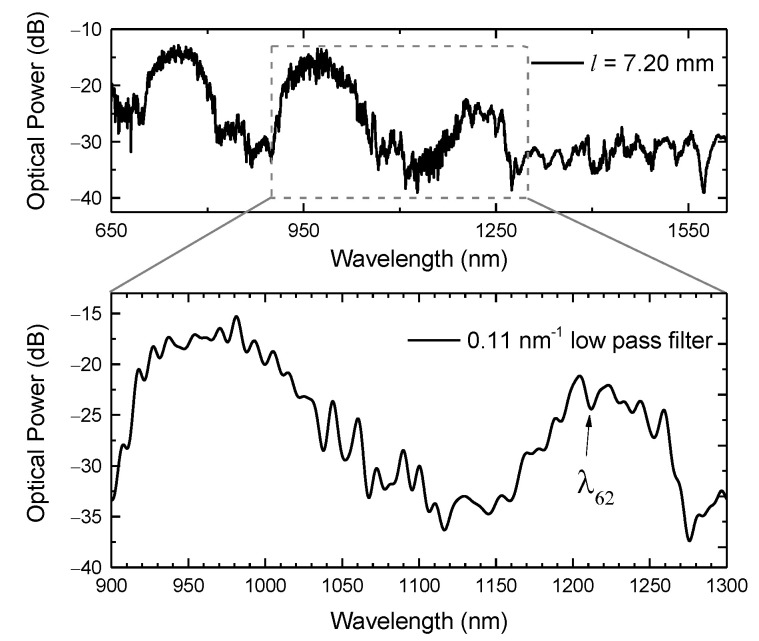
Transmission spectrum of the 7.20 mm long sensor and the respective curve attained by applying a 0.11 nm^−1^ low pass filter on the spectral range of interest. The ER wavelength (λ_62_) analyzed is also indicated in the figure.

Given all the mentioned characteristics that underlie the spectral response of the HSCF, and due to the fact that the sensor’s length does not affect the resonance characteristics, a 7.20 mm sensor was used to characterize the responses to curvature and temperature. Since one intends to resort to both IR and ER to evaluate the stablished parameters, only a broadband ranging from 900 nm to 1300 nm was monitored, where both are notoriously present. In Figure 9 is depicted the sensor spectrum in the visible and infrared windows, and in the frequency window of interest. Notice that to reduce the associated noise to the spectrum, and to obtain a more perceptible view of the ER dips, a 0.11 nm^−1^ low pass filter was applied. The experimental proceedings were performed by applying a Gaussian fit to the entire depression band (IR dip) and by monitoring the ER dip (λ_62_).

### 4.3. Sensor Characterization

**Figure 10:**
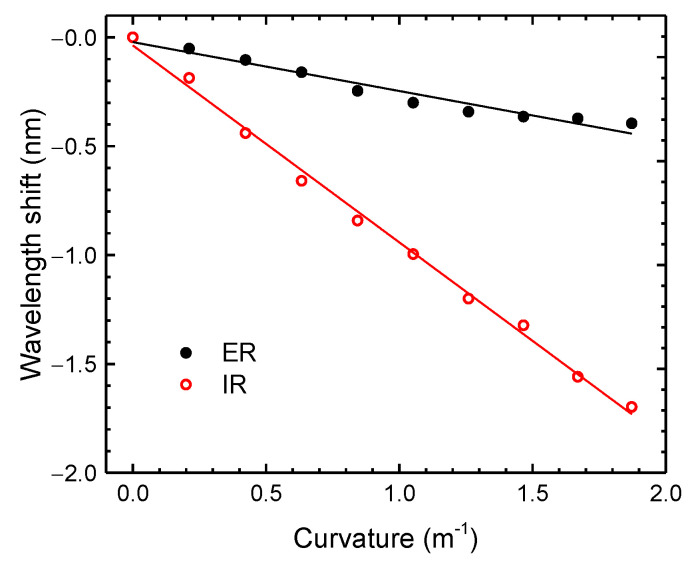
Wavelength shift dependence with the curvature of the ER and IR components.

For the curvature measurements, the distance between the fixed stages (2*L*) was set to 27.5 cm, and the sensor was bent to a maximum height (*h*) of 20 mm, corresponding to a maximum curvature of approximately 1.87 m^−1^. The decrease of height was done in steps of 2 mm. In Figure 10, the experimental results attained for the IR and ER are presented. Both components presented a shift towards smaller wavelengths (blue shift). From the results, one can infere that both resonance responses presented a linear tendency, leading to curvature sensitivities of (−0.22 ± 0.02) nm/m^−1^ (*r*^2^ = 0.95064) and (−0.90 ± 0.02) nm/m^−1^ (*r*^2^ = 0.99575), for the ER and IR, respectively. The difference between the correlation coefficients can be attributed to the low pass filter applied in the data processing. Further studies, which are not within the scope of this work, should be performed regarding the best filter to be applied in the context of an application.

When the fiber is bent, there are some phenomena that may occur. Firstly, bending causes a variation in the incident angle (*α*) of light within the fiber, which for smaller structures, such as the silica strands, will have a higher impact [48]. This can explain the higher sensitivity of the IR over the ER. On the other hand, the change in the silica refractive index due to the elasto-optic effect can also contribute to the behavior observed [49]. 

**Figure 11:**
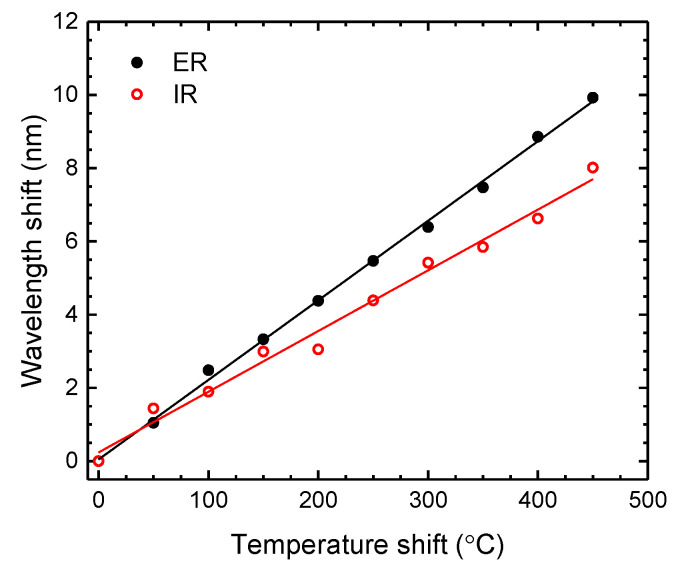
Wavelength shift dependence with temperature of the ER and IR components.

For the temperature measurements, the sensor was heated from 50 °C up to 500 °C, in steps of 50 °C. The obtained results, presented in Figure 11, show a wavelength shift toward longer wavelengths (red shift). Linear responses were attained for both components, presenting temperature sensitivities of (21.7 ± 0.3) pm/°C (*r*^2^ = 0.99809) and (16.6 ± 0.7) pm/°C (*r*^2^ = 0.98517) for ER and IR, respectively. The magnitude of these values is in agreement with what was established in the literature [50], where it is expected to achieve a higher sensitivity for the thickest resonant structure, that is, for the ER.

The presented results revealed that the sensor resonances had different responses for both measurands. The ER response (Δ*λ^ext^*) and IR response (Δ*λ^int^*) to variations on the curvature (Δ*C*) and temperature (Δ*T*) can be described by the following expressions:

(12) $$\;\lambda^{ext}\;=\;K_{C}^{ext}\cdot \Delta C\;+\;K_{T}^{ext}\cdot \Delta T,$$

(13) $$\;\lambda^{int}\;=\;K_{C}^{int}\cdot \Delta C\;+\;K_{T}^{int}\cdot \Delta T,$$

where $K_{C}^{ext}$, $K_{T}^{ext}$ are the sensitivities of the ER to curvature and temperature, respectively, and $K_{C}^{int}$ and $K_{T}^{int}$ are the respective sensitivities of the IR to curvature and temperature. Applying the definition of the matrix inversion to the Equations (12) and (13), one attains the following:

(14) $$\left[\begin{array}{c} \Delta C\\ \Delta T \end{array}\right]\;=\;\frac{1}{15.9\times 10^{-3}}\;\left[\begin{array}{cc} 0.0166 & -0.0217\\ 0.90 & -0.22 \end{array}\right]\left[\begin{array}{c} \Delta \lambda^{ext}\\ \Delta \lambda^{int} \end{array}\right].$$

Notice that the units of Δ*C*, Δ*T*, and Δ*λ* are m^−1^, °C, and nm, respectively. This outcome potentially enhances the use of this sensor in the simultaneous measurement of these parameters, making it a good candidate for several applications.

## 5. Conclusions

In conclusion, a simple configuration sensor based on an ARHCF has been presented in this work with the purpose of simultaneous measurement of curvature and temperature. The simulation analysis executed to predict the resonances behavior, inherent to the HSCF, was coherent with the experimental results and the theoretical model. Furthermore, the experimental results showed that resonance mechanisms exhibited different responses to both measurands. Curvature sensitivities of −0.90 nm/m^−1^ and −0.22 nm/m^−1^, in a curvature range of 0 nm/m^−1^ to 1.87 nm/m^−1^, were attained for the IR and ER, respectively. On the other hand, temperature sensitivities of 21.7 pm/°C and 16.6 pm/°C were respectively achieved for the ER and IR. The proposed inline sensor is innovative due to its reduced dimensions, robustness, and capability on measuring more than one parameter without needing a complex design configuration or using several sensing heads, but instead merely resorting to the ARROW guidance properties.

## Data Availability

Not applicable.
